# Accuracy of the Identification and Prognosis Prediction of SOFA-Based Sepsis-3 for Septic Patients in the Emergency Department Compared With Sepsis-2

**DOI:** 10.1155/emmi/1762179

**Published:** 2025-02-11

**Authors:** Yi-Jie Zhang, Wei Fang, Zhen Wang

**Affiliations:** Department of Emergency, Emergency and Critical Care Medical Center, Beijing Shijitan Hospital, Capital Medical University, Beijing 100038, China

**Keywords:** NEWS, prognosis, qSOFA, sepsis, SOFA

## Abstract

**Aim:** To evaluate the value of the Sequential Organ Failure Assessment (SOFA) score, a Sepsis-3 criterion, for identification and prognosis prediction among adult patients with sepsis in the emergency department (ED) compared with the Sepsis-2.

**Methods:** Adult patients with suspected sepsis presenting to the ED were retrospectively identified via Sepsis-2/Sepsis-3 criteria. The vital signs, laboratory test results, etc., were collected, and the SOFA/quick SOFA (qSOFA) scores and National Early Warning Score (NEWS) were calculated accordingly. ROC curves were generated to evaluate mortality prediction accuracy.

**Results:** Among the 481 patients included, 288/339 met the Sepsis-2/Sepsis-3 criteria, respectively, with moderate between-protocol consistency (Kappa = 0.507, *p* < 0.001; concordance = 77.3%); 115 patients (23.9%) died in hospital or within 28 days. SOFA/qSOFA scores and NEWS were significantly greater in the sepsis and death groups (*p* < 0.001), but there was no between-group difference for Sepsis-2/Sepsis-3. The temperature (T) and respiratory rate (RR) increased in the death group, whereas the systolic blood pressure (SBP) decreased. The usefulness of the SOFA score (AUC = 0.644) for predicting mortality was lower than that of qSOFA score (AUC = 0.716) and NEWS (AUC = 0.718), which could be improved (AUC = 0.701–0.721) by combining with two/three of variables (T, RR, and SBP).

**Conclusion:** Compared with Sepsis-2, Sepsis-3 identified more patients with sepsis and was suitable for ED use. The SOFA score had lower mortality prediction accuracy than the qSOFA score and NEWS, which could be significantly improved by combining with two/three variables (T, RR, and SBP).

## 1. Introduction

Sepsis and septic shock are major healthcare problems that threaten life safety worldwide. In one study, the pooled incidence was reported to be 189 hospital-treated sepsis cases per 100,000 person-years, and an estimated 26.7% of the sepsis patients died [1]. Early identification and appropriate management during the initial hours after the development of sepsis have been shown to improve outcomes [2]. To this end, international guidelines for the management of sepsis are constantly being developed. The definitions of sepsis and septic shock (Sepsis-3) were updated in 2016 by Singer et al. [3], replacing the previous definition (Sepsis-2) [4]. Sepsis-3 is defined as life-threatening organ dysfunction caused by a dysregulated host response to infection with the criterion of an acute change in the Sequential Organ Failure Assessment (SOFA) score ≥ 2 points [3].

Many large-scale studies have compared Sepsis-2 and Sepsis-3 in terms of screening and prognostic accuracy for mortality in intensive care units (ICUs) and non-ICU patients, including emergency departments (EDs) [5–8]. Despite some inconsistencies, the overall predictive value is not satisfactory. Rincon TA et al. suggested that Sepsis-2 outperforms other systems in suspected sepsis detection and is comparable to SOFA in terms of its prognostic accuracy for mortality in adult intensive care patients [5]. Moreover, several sepsis screening tools, such as systemic inflammatory response syndrome (SIRS), the quick SOFA (qSOFA) or SOFA, the National Early Warning Score (NEWS) and its derivatives, and the modified EWS (MEWS), are designed to promote early identification and prediction of severity and prognosis for sepsis and other diseases [7–10]. As shown by more studies, neither SIRS nor qSOFA, as well as the NEWS and MEWS, are ideal screening tools for sepsis in view of the limitations of each [11, 12]. The guidelines recommend against using the qSOFA as a single screening tool for sepsis or septic shock [2]. Most of the study data came from the ICU, which may have been influenced by initial treatment, and the diagnostic criteria in these studies were differentiated, especially for Sepsis-2. Most patients with potential infections, especially severe infections, first presented to the ED. The timely screening and identification of sepsis and septic shock and prediction of prognosis are crucial for emergency medical teams. In this study, we compared the usefulness of severe Sepsis-2 and Sepsis-3 to identify sepsis and predict prognosis by retrospective analysis of patients with suspected infection who presented in the ED.

## 2. Methods

This was a retrospective observational cohort study of patients with suspected infection who presented to the ED of Beijing Shijitan Hospital. All procedures adhered to the Declaration of Helsinki. The study was approved by the Ethics Review Committee of Beijing Shijitan Hospital. The requirement for consent was waived because of the retrospective nature of the study and the use of anonymized data.

### 2.1. Patients

**Figure 1:**
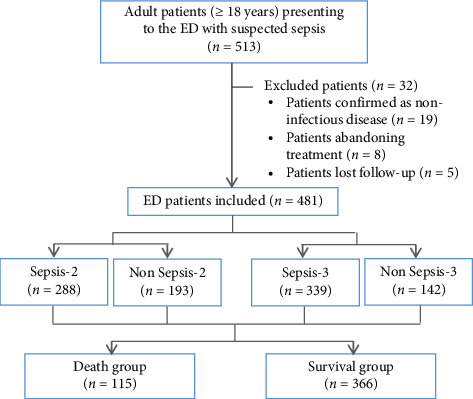
Flowchart of the study. The included and excluded patients presenting to the ED, as well as the patients in each group, are shown in the flowchart.

All 513 adult patients (aged ≥ 18 years) with suspected sepsis who presented to the ED from January 1, 2018, to December 31, 2019, were enrolled. Patients confirmed as having noninfectious disease, who abandoned treatment, who were lost to follow-up due to automatic discharge, transfer to other medical institution or other reasons were ultimately excluded. All patients were followed up for 28 days or until discharge if more than 28 days elapsed. The primary outcomes were 28-day mortality and in-hospital mortality, and all patients were divided into surviving and death groups accordingly. The flowchart is shown in Figure 1.

### 2.2. Data Collection

The data were collected from patients' electronic medical records at ED arrival, and the variables included sex, age, comorbid conditions, vital signs (temperature (T), blood pressure, heart rate (HR), respiratory rate (RR)), first laboratory test results (hemogram, bilirubin, creatinine, serum potassium and sodium concentrations, international normalized ratio (INR), activated partial thromboplastin time (APTT), lactate), first arterial blood gas analysis results, urine output, Glasgow Coma Scale (GCS), and the use of vasopressors. Accordingly, the Acute Physiology and Chronic Health Evaluation (APACHE) II, SOFA, qSOFA and NEWS scores were calculated.

The sites of infection were identified by the experts after medical record review by means of various parameters (clinical, radiological, laboratory and microbiological) and classified as follows: respiratory infection, abdominal infection, urogenital infection, skin/soft tissue infection, central nerve infection, and hematologic infection. The comorbid conditions included diabetes mellitus, chronic pulmonary disease, renal failure, cerebrovascular disease, congestive heart failure, malignant tumor, connective tissue disease, liver disease, and hematopoietic disease. The Charlson Comorbidity Index (CCI) was calculated on the basis of comorbidities [13].

### 2.3. Clinical Criteria

Sepsis diagnosis was identified on the basis of the required criteria and score. In brief, Sepsis-2 is defined as severe sepsis (sepsis with organ dysfunction or tissue hypoperfusion) meeting one or more of the following criteria: arterial hypoxemia (PaO_2_/FIO_2_ < 300), acute oliguria (urine output < 0.5 mL·kg^−1^ hr^−1^ or 45 mmol/L for at least 2 h), creatinine increase > 0.5 mg/dL, coagulation abnormalities (INR > 1.5 or APTT > 60 s), ileus (absent bowel sounds), thrombocytopenia (platelet count < 100,000/μL), hyperbilirubinemia (plasma total bilirubin > 4 mg/dL or 70 mmol/L), hyperlactatemia (> 1 mmol/L), decreased capillary refill or mottling [4]. Sepsis-3 is identified as an acute change in the total SOFA score ≥ 2 points consequent to infection [3]. The SOFA scores were acquired at the ED arrival and ranged from 0 to 24. Missing values were presumed to be normal and contributed zero points to the scores.

### 2.4. Statistical Analysis

The data are presented as the means ± SDs for normally distributed continuous variables or as median with interquartile ranges (IQRs) if not normally distributed. Statistical comparisons between two groups were performed using the two-tailed Student's *t*-test for normally distributed continuous variables or the Mann–Whitney *U* test for nonnormally distributed continuous variables. Categorical data are presented as frequencies and percentages and were compared using the chi-square test. Cohen's kappa test was used to assess concordance between the Sepsis-2 and Sepsis-3 diagnostic criteria. Receiver operating characteristic (ROC) curves were constructed, and the area under the ROC curve (AUC) was calculated to assess the predictive power. The DeLong test was used to compare the significant differences between the ROC curves. The predictive value was classified on the basis of the AUC as excellent discrimination (AUC value: 0.99–0.9), very good discrimination (AUC value: 0.89–0.8), good (AUC value: 0.79–0.7), moderate (AUC value: 0.69–0.6), or poor (AUC value: < 0.6) [14]. Odds ratios (ORs) and their 95% confidence intervals (CIs) were estimated using logistic regression to determine the associations of the indicators with mortality risk. Statistical significance was set at *p* < 0.05. The data were analyzed using SPSS statistical software (Version 27.0; IBM, Armonk, New York).

## 3. Results

### 3.1. General Patient Characteristics

A total of 481 ED patients (305 men and 176 women) with confirmed infection were included, among whom 288 patients met the criteria for Sepsis-2, 339 patients met the criteria for Sepsis-3, and 259 patients met the criteria for both Sepsis-2 and Sepsis-3. Cohen's kappa test revealed that the consistency of the two diagnoses protocols was moderate (Kappa = 0.507, *p* < 0.001; concordance = 77.3%). As shown in Table 1, compared with the Sepsis-2 group, more patients were enrolled in the Sepsis-3 group with significantly more males than females. The age of all the patients ranged from 18 to 101 years, with no statistically significant difference between the groups. Higher body T and lower systolic blood pressure (SBP) (*p* < 0.001) were observed in patients with sepsis than in those without sepsis, but there was no difference between the Sepsis-2 and Sepsis-3 groups. Among all patients, 115 (23.91%) died in the hospital or within 28 days, including 82 (28.47%) in the Sepsis-2 group and 97 (28.61%) in the Sepsis-3 group, with no significant difference in mortality between the two groups.

In terms of the infection sites listed in Table 1, respiratory infection accounted for the highest proportion (89.81%), followed by hematologic (15.59%), urogenital (11.64%), and abdominal infection (5.20%). Notably, compared with the nonsepsis group, the Sepsis-2 and Sepsis-3 groups had more patients with respiratory, urogenital, and abdominal infections (all *p* < 0.05). However, the sites of infection were not significantly different between the Sepsis-2 and Sepsis-3 groups. Microbial cultures of sputum, blood, urine, pleural fluid, abdominal fluid, etc., were obtained from 397 patients, 69 of whom had positive blood culture results. The results revealed that more patients with positive blood cultures were in the sepsis groups than in the nonsepsis group, but there was no significant difference between the Sepsis-2 and Sepsis-3 groups.

Among all the patients, 295 (61.33%) had one or more comorbidities, and the specific classification and proportion are shown in Table 1. Overall, the difference in the CCI was not statistically significant among the groups.

The statistical results of the surviving and death groups are presented in Table 2. The age, T, and RR of patients at ED arrival in the death group were significantly greater than those in the surviving group (*p* < 0.01), whereas the SBP was lower in the death group (*p* < 0.001). There were no significant differences in sex, HR, or CCI between the two groups.

### 3.2. Scoring Systems

Five scoring systems, the APACHE II, GCS, SOFA, qSOFA, and NEWS, were included in this study for rapid screening and severity assessment of sepsis. As shown in Table 1, all the scores in the sepsis group were significantly greater than those in the nonsepsis group, but there was no significant difference between the Sepsis-2 and Sepsis-3 groups. In addition, compared with those in the surviving group, all scoring systems shown in Table 2 were increased in the death group (all *p* < 0.001).

### 3.3. ROC Curve and Logistic Regression Analysis

**Figure 2:**
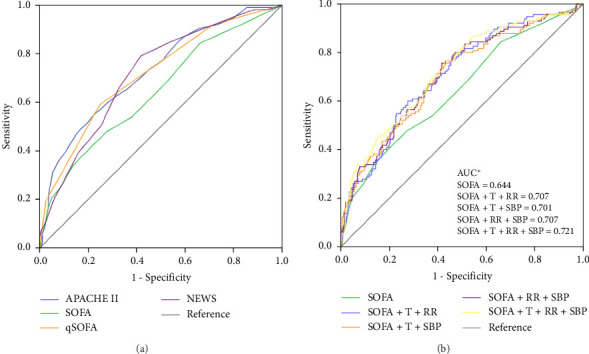
ROC curve analysis: (a) ROC curves for the Acute Physiology and Chronic Health Evaluation (APACHE) II, Sequential Organ Failure Assessment (SOFA), quick SOFA (qSOFA), and National Early Warning Score (NEWS). (b) ROC curves and AUCs for the SOFA score and SOFA score combined with two or three of temperature (T), respiratory rate (RR), and systolic blood pressure (SBP) data. ^∗^*p* ≤ 0.01: SOFA score versus SOFA score combined with two or three of T, RR, and SBP (DeLong test).

**Figure 3:**
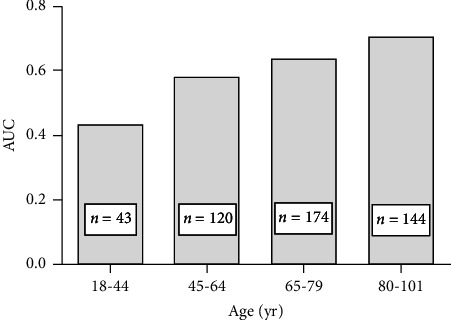
AUCs of the SOFA score across different age intervals.

ROC curve (Figure 2 and Table 3) and logistic regression analyses were used to evaluate the ability of the scoring system to predict poor prognosis, especially the SOFA score, which is the criterion for Sepsis-3. The results illustrated in Figure 2(a) indicate that the usefulness of the SOFA score (AUC value 0.644) for predicting mortality was moderate, which was lower than that of the APACHE II score, qSOFA score, and NEWS (*p* ≤ 0.01). The cutoff values, sensitivity, and specificity are provided in Table 3. Moreover, when the SOFA score was ≥ 2, the sensitivity for predicting mortality was high (84.3%), but the specificity was poor (33.9%). In contrast, a qSOFA score ≥ 2 had low sensitivity (59.1%) and high specificity (75.1%) in predicting mortality. By exploring the possible factors influencing the SOFA score, we found that the prognostic value of the SOFA score in ED patients with sepsis tended to increase with increasing age (Figure 3) and was good in the elderly patients (≥ 80 years) and poor in young and middle-aged patients.

According to the logistic regression analysis (Table 4), the SOFA score, age, T, and RR were positively correlated with poor prognosis, whereas SBP was negatively correlated. To improve the predictive ability of the SOFA score, ROC curves were generated by combining the SOFA score with one or more factors such as age, T, RR, and SBP according to the simplest principle. The results (Figure 2(b)) revealed that the usefulness of the SOFA score for predicting mortality was significantly improved when it was combined with two or all of T, RR, and SBP (AUC value 0.701–0.721, *p* ≤ 0.01). The DeLong test showed that the prognostic ability of the modified SOFA score was similar to that of the APACHE II score, qSOFA score, and NEWS (*p* ≥ 0.05).

## 4. Discussion

With improved understanding of sepsis pathobiology, the definition of sepsis was updated as Sepsis-3, which focuses more on organ dysfunction caused by a dysregulated host response to infection, which is associated with an in-hospital mortality greater than 10% [3]. Sepsis-3 unified diagnostic criteria and the acute change in total SOFA score ≥ 2 points consequent to the infection are confirmed as the essential criterion for screening and identifying Sepsis-3. Since then, many studies have compared the use of the Sepsis-2 and Sepsis-3 criteria in screening and predicting prognosis, mainly for ICU and hospitalized patients. More notably, the diagnostic criteria for Sepsis-2 used by different studies are inconsistent, especially for severe sepsis [5–8, 15]. Therefore, the conclusions are not entirely consistent. In this study, the criteria for severe sepsis (sepsis with organ dysfunction) in Sepsis-2 proposed by Levy MM et al. in 2003 were adopted and were comparable to those of Sepsis-3. We focused on patients with suspected sepsis presented in the ED to further evaluate the screening ability and prognostic accuracy of Sepsis-2 and Sepsis-3 and the application value of the SOFA score in the ED.

More patients were identified on the basis of Sepsis-3 than Sepsis-2 with similar mortality.

To date, no gold standard for sepsis has been established. The items in the criteria are identified on the basis of their validity and usefulness in the clinic. Despite inconsistent diagnostic criteria, previous studies have reported good concordance of 81%–92% in diagnoses based on Sepsis-2 and Sepsis-3 for adult ICU patients in Japan, Brazil, and England [6, 16, 17]. In this study, 89.9% of the patients diagnosed with Sepsis-2 in the ED met the criteria for Sepsis-3. However, the concordance of diagnoses based on severe sepsis in Sepsis-2 and Sepsis-3 was 77.3%, which was slightly lower than that reported in previous studies. This discrepancy is attributed mainly to the different compositions of the participants and inconsistent diagnostic criteria for Sepsis-2. Moreover, we noted that Sepsis-3 screened more patients than Sepsis-2 did, and there were no significant differences in mortality, age, vital signs, comorbidities, site of infection, or scoring systems between the two groups. According to previous studies, the observed mortality of sepsis varies greatly from 18.7% to 64.5% [5, 8, 16, 18], which is likely related to different economic levels and overall medical conditions in different countries. Some studies revealed that patients meeting the criteria for Sepsis-3 have a greater risk of mortality than those meeting the criteria for Sepsis-1 and Sepsis-2 [6, 19, 20], but no such difference was observed in our study. In addition to the reasons mentioned above, whether this discrepancy is related to the existence of more uncertain factors affecting the accuracy of the SOFA score during emergencies remains to be further explored.

The qSOFA score is not considered an ideal screening tool due to its high specificity and low sensitivity for the early identification of infection-induced organ dysfunction [2, 12, 21]. Seymour CW et al. reported that 24% of the infected patients had a qSOFA score of 2 or 3, but these patients accounted for 70% of poor outcomes [22]. In this study, patients with qSOFA ≥ 2 accounted for only 42.36% of Sepsis-2 diagnoses and 41% of the Sepsis-3 diagnoses, which once again proved that the qSOFA score is not suitable as a screening tool for sepsis in ED patients.

The accuracy of the SOFA score in predicting the mortality of sepsis patients was inferior to that of the qSOFA score and NEWS and can be improved by combining with two or three variables (T, RR, and SBP).

In previous studies, the SOFA score, a diagnostic criterion for Sepsis-3, has been shown to be less accurate in predicting the prognosis of sepsis than early warning systems, such as the qSOFA score and NEWS [6, 7], which is consistent with the results of this study. In this study, SOFA score ≥ 2 exhibited high sensitivity and poor specificity in predicting mortality. Notably, the low sensitivity of the qSOFA score as a predictor of poor prognosis in sepsis, as in this study, should also cause alarm, despite its high specificity [6, 7, 22, 23]. Parks Taylor S et al. reported that the accuracy of qSOFA for predicting in-hospital mortality diminished with increasing comorbidity burden [24], which may limit its clinical application [23]. Moreover, the NEWS is widely used in the UK as a risk stratification tool for acutely ill patients with sepsis [25]. However, its prognostic accuracy has been inconsistent across studies [11, 25, 26]. In a multicenter study including 773,477 hospitalized patients in the ED or a non-ICU hospital ward, the NEWS presented the highest discrimination, which was clearly superior to MEWS, Between the Flags (BTFs), qSOFA, and SIRS, and was suggested to identify patients with and without infection at high risk of mortality [11]. In contrast, some studies have reported that the NEWS is sensitive but not specific [25], and its accuracy is inferior to that of the qSOFA [26]. A scoring system with ideal sensitivity and specificity for predicting the prognosis of sepsis is still lacking. Moreover, experts have focused on how to improve the accuracy of the SOFA score in predicting the prognosis of sepsis. Raith EP et al. reported that an increase in the SOFA score of 2 or more had greater prognostic accuracy for in-hospital mortality than SIRS criteria or the qSOFA score [8]. Another study revealed that incorporating SIRS and SOFA criteria into the same model can improve the discrimination of mortality [27]. According to the ROC curve analysis results of this study, the capacity of the qSOFA score to predict prognosis was good and similar to that of the NEWS and that of the SOFA score was moderate. The predictive ability of the SOFA score was affected by age. The poor predictive ability in patients < 65 years decreased the overall predictive ability. Notably, we found that the ability of the SOFA score can be significantly improved by combining with two or three simple and readily available indicators (T, RR, and SBP). The modified SOFA score exhibited comparable efficacy to the qSOFA score and NEWS in predicting the mortality of patients with sepsis in ED, which is worthy of further study.

This study was conducted in a relatively large group of patients; however, it has several limitations. This study was a retrospective observational study from a single center at a tertiary hospital in a developing country, which needs to be confirmed by further multicenter prospective studies with larger samples.

## 5. Conclusion

In this study, we evaluated the effects of changes in the definition of sepsis on the ability to screen and predict mortality in ED patients by comparing severe sepsis in Sepsis-2 and Sepsis-3 at comparable levels. The results revealed that compared with Sepsis-2, Sepsis-3 could identify more patients with sepsis with similar mortality. Sepsis-3 is confirmed to be more suitable in the ED, and its use can increase the number of high-risk patients with sepsis receiving timely treatments. SOFA, as the diagnostic criterion for Sepsis-3, had a lower accuracy in predicting mortality than the qSOFA score and NEWS, but its accuracy can be significantly improved by combining it with two or three other items (T, RR, and SBP).

## Figures and Tables

**Table 1:** Demographic, clinical data at arrival and outcomes for all, Sepsis-2, and Sepsis-3 patients, respectively.

Parameter	All (*n* = 481)	Sepsis-2 (*n* = 288)	Non Sepsis-2 (*n* = 193)	*p* value	Sepsis-3 (*n* = 339)	Non Sepsis-3 (*n* = 142)	*p* value	*p* value (Sepsis-2 vs. Sepsis-3)
Sex								
Male *n* (%)	305 (63.41)	181 (62.85)	124 (64.25)	0.754	225 (66.37)	80 (56.34)	0.037	0.357
Age yr. mean (min, max)	68.74 (18, 101)	69.66 (21, 101)	67.37 (18, 98)	0.075	69.05 (18, 101)	68.01 (18, 94)	0.469	0.569
Vital sign mean ± SD								
T (°C)	37.36 ± 0.95	37.49 ± 0.97	37.18 ± 0.89	< 0.001	37.46 ± 0.98	37.12 ± 0.83	< 0.001	0.779
HR (bpm)	101.41 ± 19.68	101.73 ± 21.07	100.92 ± 17.43	0.648	101.31 ± 19.67	101.64 ± 19.75	0.865	0.795
RR (bpm)	24.27 ± 6.82	24.63 ± 6.91	23.74 ± 6.66	0.158	24.40 ± 6.76	23.97 ± 6.98	0.532	0.669
SBP (mmHg)	123.86 ± 28.03	118.13 ± 28.04	132.39 ± 25.83	< 0.001	120.64 ± 27.55	131.51 ± 27.78	< 0.001	0.259
Site of infection *n* (%)								
Respiratory	432 (89.81)	248 (86.11)	184 (95.34)	0.001	297 (87.61)	135 (95.07)	0.014	0.579
Abdominal	25 (5.20)	20 (6.94)	5 (2.59)	0.035	22 (6.49)	3 (2.11)	0.049	0.820
Urogenital	56 (11.64)	43 (14.93)	13 (6.74)	0.006	48 (14.16)	8 (5.63)	0.008	0.785
Skin/soft tissue	6 (1.25)	3 (1.04)	3 (1.55)	0.938	4 (1.18)	2 (1.41)	1.000	0.870
Central nerve	11 (2.29)	9 (3.13)	2 (1.04)	0.234	10 (2.95)	1 (0.70)	0.243	0.899
Hematologic	75 (15.59)	63 (21.88)	12 (6.22)	< 0.001	63 (18.58)	12 (8.45)	0.133	0.358
Positive blood culture *n* (%)	69 (14.35)	59 (20.48)	10 (5.18)	< 0.001	58 (17.11)	11 (7.75)	0.008	0.289
Comorbid conditions *n* (%)	295 (61.33)	180 (62.50)	115 (59.59)	0.520	200 (59.00)	95 (66.90)	0.104	0.371
Diabetes mellitus	107 (22.25)	69 (23.96)	38 (19.69)	0.270	73 (21.53)	34 (23.94)	0.562	0.470
Chronic pulmonary disease	95 (19.75)	44 (15.28)	51 (26.42)	0.003	52 (15.34)	43 (30.28)	< 0.001	0.983
Renal failure	66 (13.72)	53 (18.40)	13 (6.74)	< 0.001	63 (18.58)	3 (2.11)	< 0.001	0.954
Cerebrovascular disease	60 (12.47)	33 (11.46)	27 (13.99)	0.410	47 (13.86)	13 (9.15)	0.154	0.368
Congestive heart failure	36 (7.48)	24 (8.33)	12 (6.22)	0.387	25 (7.37)	11 (7.75)	0.888	0.656
Malignant tumor	32 (6.65)	19 (6.60)	13 (6.74)	0.952	20 (5.90)	12 (8.45)	0.306	0.719
Connective tissue disease	9 (1.87)	4 (1.39)	5 (2.59)	0.542	3 (0.88)	6 (4.22)	0.036	0.828
Liver disease	8 (1.66)	5 (1.74)	3 (1.55)	1.000	5 (1.47)	3 (2.11)	0.914	1.000
Hematopoietic disease	8 (1.66)	8 (2.78)	0 (0)	0.049	7 (2.06)	1 (0.70)	0.501	0.560
CCI median (IQR)	1 (0, 2)	1 (0, 2)	1 (0, 1)	0.299	1 (0, 2)	1 (0, 1)	0.391	0.870
Scoring system								
APACHE II mean (min, max)	15.75 (1, 40)	17.83 (2, 40)	12.65 (1, 29)	< 0.001	17.59 (1, 40)	11.37 (2, 29)	< 0.001	0.678
GCS mean (min, max)	13.04 (3, 15)	12.60 (3, 15)	13.70 (3, 15)	< 0.001	12.37 (3, 15)	14.65 (3, 15)	< 0.001	0.365
SOFA ≥ 2 *n* (%)	339 (70.48)	259 (89.93)	80 (41.45)	< 0.001	339 (100)	0 (0)	< 0.001	0.923
qSOFA ≥ 2 *n* (%)	158 (32.85)	122 (42.36)	36 (18.65)	< 0.001	139 (41.00)	19 (13.38)	< 0.001	0.753
NEWS mean (min, max)	5.95 (0, 17)	7.09 (0, 17)	4.26 (0, 14)	< 0.001	6.89 (0, 17)	3.71 (0, 12)	< 0.001	0.420
Mortality *n* (%)	115 (23.91)	82 (28.47)	33 (17.10)	0.004	97 (28.61)	18 (12.68)	< 0.001	0.969

Abbreviations: APACHE, Acute Physiology and Chronic Health Evaluation; CCI, Charlson Comorbidity Index; GCS, Glasgow Coma Scale; HR, heart rate; NEWS, National Early Warning Score; qSOFA, quick SOFA; RR, respiratory rate; SBP, systolic blood pressure; SOFA, Sequential Organ Failure Assessment; T, temperature.

**Table 2:** Demographic, vital signs, CCI, and scoring systems in the surviving and death groups.

Parameter	Surviving group (*n* = 366)	Death group (*n* = 115)	*p* value
Sex			
Male *n* (%)	234 (63.93)	71 (61.74)	0.670
Age yr. mean (min, max)	67.73 (18, 101)	71.97 (21, 96)	0.009
Vital sign mean ± SD			
T (°C)	37.25 ± 0.95	37.72 ± 0.84	< 0.001
HR (bpm)	101.09 ± 20.23	102.42 ± 17.85	0.528
RR (bpm)	23.58 ± 7.07	26.47 ± 5.43	< 0.001
SBP (mmHg)	127.19 ± 27.51	113.28 ± 27.14	< 0.001
CCI median (IQR)	1 (0, 2)	1 (0, 2)	0.120
Scoring system median (IQR)			
APACHE II	14 (9, 19)	21 (14, 26)	< 0.001
GCS	15 (14, 15)	15 (9, 15)	< 0.001
SOFA	3 (1, 5)	4 (2, 8)	< 0.001
qSOFA	1 (0, 1.25)	2 (1, 2)	< 0.001
NEWS	5 (3, 8)	8 (6, 10)	< 0.001

Abbreviations: APACHE, acute physiology and chronic health evaluation; CCI, Charlson Comorbidity Index; GCS, Glasgow Coma Scale; HR, heart rate; NEWS, National Early Warning Score; qSOFA, quick SOFA; RR, respiratory rate; SBP, systolic blood pressure; SOFA, Sequential Organ Failure Assessment; T, temperature.

**Table 3:** ROC curve analysis for predicting mortality.

Parameter	AUC	95% CI	Cutoff	Sensitivity	Specificity	Youden's index
APACHE II	0.727	0.674–0.780	19.50	0.557	0.768	0.324
SOFA⁣^∗^	0.644	0.585–0.703	5.50	0.409	0.795	0.204
qSOFA	0.716	0.662–0.770	1.50	0.591	0.751	0.343
NEWS	0.718	0.666–0.770	5.50	0.791	0.582	0.373

Abbreviations: APACHE, Acute Physiology and Chronic Health Evaluation; NEWS, National Early Warning Score; qSOFA, quick SOFA; SOFA, Sequential Organ Failure Assessment.

^∗^ *p* ≤ 0.01: SOFA versus APACHE II, qSOFA, or NEWS (DeLong test).

**Table 4:** Logistics regression analysis base on mortality.

	SOFA	Age (yr.)	T (°C)	RR (bpm)	SBP (mmHg)
OR	1.12	1.02	1.43	1.05	0.99
95% CI	1.04–1.20	1.01–1.04	1.12–1.82	1.02–1.09	0.98–0.99
*p* value	0.003	0.003	0.004	0.003	0.001

Abbreviations: RR, respiratory rate; SBP, systolic blood pressure; SOFA, sequential organ failure assessment; T, temperature.

## Data Availability

The data that support the findings of this study are available from the corresponding author upon reasonable request.

## References

[1] Fleischmann-Struzek C., Mellhammar L., Rose N. (2020). Incidence and Mortality of Hospital- and ICU-Treated Sepsis: Results from an Updated and Expanded Systematic Review and Meta-Analysis. *Intensive Care Medicine*.

[2] Evans L., Rhodes A., Alhazzani W. (2021). Surviving Sepsis Campaign: International Guidelines for Management of Sepsis and Septic Shock 2021. *Intensive Care Medicine*.

[3] Singer M., Deutschman C. S., Seymour C. W. (2016). The Third International Consensus Definitions for Sepsis and Septic Shock (Sepsis-3). *JAMA*.

[4] Levy M. M., Fink M. P., Marshall J. C. (2003). 2001 SCCM/ESICM/ACCP/ATS/SIS International Sepsis Definitions Conference. *Critical Care Medicine*.

[5] Rincon T. A., Raffa J., Celi L. A. (2023). Evaluation of Evolving Sepsis Screening Criteria in Discriminating Suspected Sepsis and Mortality Among Adult Patients Admitted to the Intensive Care Unit. *International Journal of Nursing Studies*.

[6] Machado Lessa C. L., Branchini G., Moreira Delfino I. (2024). Comparison of Sepsis-1, 2 and 3 for Predicting Mortality in Septic Patients of a Middle-Income Country: A Retrospective Observational Cohort Study. *Journal of Intensive Care Medicine*.

[7] Freund Y., Lemachatti N., Krastinova E. (2017). Prognostic Accuracy of Sepsis-3 Criteria for In-Hospital Mortality Among Patients with Suspected Infection Presenting to the Emergency Department. *JAMA*.

[8] Raith E. P., Udy A. A., Bailey M. (2017). Prognostic Accuracy of the SOFA Score, SIRS Criteria, and qSOFA Score for In-Hospital Mortality Among Adults with Suspected Infection Admitted to the Intensive Care Unit. *JAMA*.

[9] Islam M. M., Nasrin T., Walther B. A., Wu C. C., Yang H. C., Li Y. C. (2019). Prediction of Sepsis Patients Using Machine Learning Approach: A Meta-Analysis. *Computer Methods and Programs in Biomedicine*.

[10] Zhang Y. J., Liu X. Y., Xu W. X., Yang Y. P. (2024). Reevaluation of Prognostic and Severity Indicators for COVID-19 Patients in the Emergency Department. *Annals of Medicine*.

[11] Liu V. X., Lu Y., Carey K. A. (2020). Comparison of Early Warning Scoring Systems for Hospitalized Patients with and without Infection at Risk for In-Hospital Mortality and Transfer to the Intensive Care Unit. *JAMA Network Open*.

[12] Serafim R., Gomes J. A., Salluh J., Póvoa P. (2018). A Comparison of the Quick-SOFA and Systemic Inflammatory Response Syndrome Criteria for the Diagnosis of Sepsis and Prediction of Mortality: A Systematic Review and Meta-Analysis. *Chest*.

[13] Charlson M. E., Carrozzino D., Guidi J., Patierno C. (2022). Charlson Comorbidity Index: A Critical Review of Clinimetric Properties. *Psychotherapy and Psychosomatics*.

[14] Nahm F. S. (2022). Receiver Operating Characteristic Curve: Overview and Practical Use for Clinicians. *Korean journal of anesthesiology*.

[15] Eriksson J., Eriksson M., Brattström O. (2019). Comparison of the Sepsis-2 and Sepsis-3 Definitions in Severely Injured Trauma Patients. *Journal of Critical Care*.

[16] Abe T., Yamakawa K., Ogura H. (2020). Epidemiology of Sepsis and Septic Shock in Intensive Care Units between Sepsis-2 and Sepsis-3 Populations: Sepsis Prognostication in Intensive Care Unit and Emergency Room (SPICE-ICU). *Journal of intensive care*.

[17] Shankar-Hari M., Harrison D. A., Rubenfeld G. D., Rowan K. (2017). Epidemiology of Sepsis and Septic Shock in Critical Care Units: Comparison between Sepsis-2 and Sepsis-3 Populations Using a National Critical Care Database. *British Journal of Anaesthesia*.

[18] Quintano Neira R. A., Hamacher S., Japiassú A. M. (2018). Epidemiology of Sepsis in Brazil: Incidence, Lethality, Costs, and Other Indicators for Brazilian Unified Health System Hospitalizations from 2006 to 2015. *PLoS One*.

[19] Khwannimit B., Bhurayanontachai R., Vattanavanit V. (2018). Comparison of the Performance of SOFA, qSOFA and SIRS for Predicting Mortality and Organ Failure Among Sepsis Patients Admitted to the Intensive Care Unit in a Middle-Income Country. *Journal of Critical Care*.

[20] Driessen R. G. H., van de Poll M. C. G., Mol M. F., van Mook W., Schnabel R. M. (2018). The Influence of a Change in Septic Shock Definitions on Intensive Care Epidemiology and Outcome: Comparison of Sepsis-2 and Sepsis-3 Definitions. *Infectious diseases (London, England)*.

[21] Herwanto V., Shetty A., Nalos M. (2019). Accuracy of Quick Sequential Organ Failure Assessment Score to Predict Sepsis Mortality in 121 Studies Including 1,716,017 Individuals: A Systematic Review and Meta-Analysis. *Critical care explorations*.

[22] Seymour C. W., Liu V. X., Iwashyna T. J. (2016). Assessment of Clinical Criteria for Sepsis: For the Third International Consensus Definitions for Sepsis and Septic Shock (Sepsis-3). *JAMA*.

[23] Jiang J., Yang J., Mei J., Jin Y., Lu Y. (2018). Head-to-head Comparison of qSOFA and SIRS Criteria in Predicting the Mortality of Infected Patients in the Emergency Department: a Meta-Analysis. *Scandinavian Journal of Trauma, Resuscitation and Emergency Medicine*.

[24] Parks Taylor S., McWilliams A., Taylor B. T. (2019). Predictive Accuracy of Quick Sequential Organ Failure Assessment for Hospital Mortality Decreases with Increasing Comorbidity Burden Among Patients Admitted for Suspected Infection. *Critical Care Medicine*.

[25] Almutary A., Althunayyan S., Alenazi K. (2020). National Early Warning Score (NEWS) as Prognostic Triage Tool for Septic Patients. *Infection and Drug Resistance*.

[26] Wang C., Xu R., Zeng Y., Zhao Y., Hu X. (2022). A Comparison of qSOFA, SIRS and NEWS in Predicting the Accuracy of Mortality in Patients with Suspected Sepsis: A Meta-Analysis. *PLoS One*.

[27] Engoren M., Seelhammer T., Freundlich R. E., Maile M. D., Sigakis M. J. G., Schwann T. A. (2020). A Comparison of Sepsis-2 (Systemic Inflammatory Response Syndrome Based) to Sepsis-3 (Sequential Organ Failure Assessment Based) Definitions-A Multicenter Retrospective Study. *Critical Care Medicine*.

